# *In situ* synthesis of MoS_2_/graphene nanosheets as free-standing and flexible electrode paper for high-efficiency hydrogen evolution reaction†

**DOI:** 10.1039/c8ra01226a

**Published:** 2018-03-16

**Authors:** Xianghui Zhang, Mingguang Zhang, Yiqun Tian, Jing You, Congxing Yang, Jun Su, Yuebin Li, Yihua Gao, Haoshuang Gu

**Affiliations:** Hubei Collaborative Innovation Center for Advanced Organic Chemical Materials, Hubei Key Laboratory of Ferro & Piezoelectric Materials and Devices, Faculty of Physics & Electronic Sciences, Hubei University Wuhan 430062 P. R. China xhzhang@hubu.edu.cn guhsh@hubu.edu.cn; Center for Nanoscale Characterization and Devices, School of Physics, Wuhan National Laboratory for Optoelectronics, Huazhong University of Science and Technology Luoyu Road 1037 Wuhan 430074 P. R. China

## Abstract

In this article, an exquisite flexible hybrid MoS_2_/graphene free-standing electrocatalyst paper was fabricated by a one-step *in situ* solvothermal process. The assembled MoS_2_/graphene catalysts exhibit significantly enhanced electrocatalytic activity and cycling stability towards the splitting of water in acidic solution. Furthermore, a strategic balance of abundant active sites at the edge of the S–Mo–S layers with efficient electron transfer in the MoS_2_/graphene hybrid catalyst plays a key role in controlling the electrochemical performance of the MoS_2_ nanosheets. Most importantly, the hybrid MoS_2_/graphene nanosheet paper shows excellent flexibility and high electrocatalytic performance under the various bending states. This work demonstrates an opportunity for the development of flexible electrocatalysts, which have potential applications in renewable energy conversion and energy storage systems.

## Abstract 2

An improved flexible hybrid MoS_2_/graphene free-standing electrocatalyst paper was fabricated by a one-step *in situ* solvothermal process for hydrogen evolution reaction applications.

## Graphical abstract

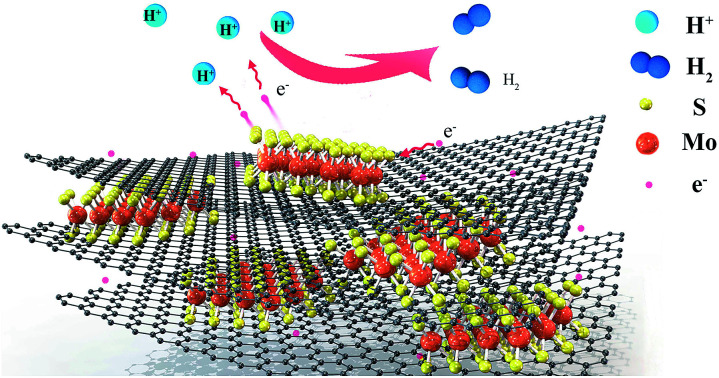

## Introduction

To address the issues of the global energy crisis and environmental contamination, researchers have devoted considerable attention to hydrogen (H_2_) as a renewable and environmentally-friendly energy source to replace the current hydrocarbon fuels. One possible route to target is to utilize solar energy directly or indirectly (through wind power, for instance) to split water to produce H_2_.^1^ Presently, producing H_2_ from the electrocatalytic splitting of water using the hydrogen evolution reaction (HER) holds tremendous promise because of the high energy efficiency and the economical and practical advantages.^2^ An advanced catalyst for the HER should play an important role in reducing the overpotential and consequently increasing the efficiency of this electrochemical process. Although the most effective HER electrocatalysts are Pt or Pt-based materials, their high cost and low abundance substantially restrict their large-scale application. Thus, it remains challenging to develop highly active HER catalysts based on non-noble-metal materials that are more abundant and lower cost.^3–8^

Molybdenum disulfide (MoS_2_) is a layered transition metal dichalcogenide (TMD), which has triggered broad attention as a typical two-dimensional (2D) semiconductor nanomaterial. The structure of MoS_2_ is based on a hexagonal crystal, where each Mo atom is six-fold coordinated, hexagonally packed between two three-fold coordinated sulfur atoms. Each S–Mo–S layer is weakly bonded to other S–Mo–S layers by van der Waals forces. Recent works showed MoS_2_ to be a promising electrocatalyst for the HER.^9–15^ It has been suggested that the electrocatalyst activation originates from the unsaturated sulfur atoms at the edges of the MoS_2_ plates, while the basal planes were often thought to be catalytically inert with some exceptions. As a result, nanosized MoS_2_ with a high degree of exposed edges should be more active for HER electrocatalysis than materials in bulk forms.^16–18^ Enormous effort has been dedicated to exposing more edge sites to enhance MoS_2_ HER activity by engineering various morphologies, such as nanoparticles,^19^ nanoplates,^20^ nanosheets,^21,22^ nanoflowers,^23^ nanoboxes,^24,25^ nanoporous films^26^ and heterolayer nanowires.^27^ The electrical conductivity of catalysts is another crucial factor affecting the electrocatalytic activity because high conductivity ensures fast electron transport during the catalytic process. However, poor electrical conductivity often exists both in dispersed nanostructure MoS_2_ catalysts and between the S–Mo–S layers of MoS_2_, restricting the activity.^28^ Considerable attention has been focused on accelerating electron transfer by coupling MoS_2_ with conductive substrates, such as nanoporous gold,^29,30^ 3D Ni foam,^31^ metal oxides,^32–34^ activated carbon,^35^ carbon fibers,^36,37^ and carbon nanotubes (CNTs).^38,39^ Among these conductive supports, graphene, particularly reduced graphene oxide (RGO), has attracted much attention because of its excellent electron transport properties, chemical stability and solution-phase processing compatibility.^40,41^ Therefore, graphene supported MoS_2_ nanostructures appear to be highly active and stable electrocatalysts. However, all of the MoS_2_/graphene hybrid nanostructure catalysts mentioned above were prepared in powdered form. Thus they had to be dropped onto the surface of a conductive substrate (*e.g.* a glassy carbon electrode) and then be immobilized by binding Nafion or PTFE (polytetrafluoroethylene) polymer as a working electrode. As a result, the binding polymer might block some catalytically active sites, increase the series resistance, and thus decrease the overall activity of the material. For this reason, binder-free HER catalysts based on graphene with high electrocatalytic performance are highly interesting. On the other hand, an appropriate loading rate of MoS_2_ onto the graphene nanosheet is one of the significant parameters to study in MoS_2_/graphene hybrid electrocatalysts to balance abundant active sites at the edge of S–Mo–S layers with efficient electron transfer. However, so far there have been almost no reports on the activity of MoS_2_/graphene hybrid electrocatalysts sensitive to the MoS_2_ nanostructure loading rate.

Herein, we synthesize MoS_2_/graphene hybrid nanosheets by a one-step *in situ* solvothermal method and fabricate a free-standing electrocatalyst paper for the HER to address the above mentioned problems. The MoS_2_/graphene paper exhibits excellent flexibility, high conductivity, a large surface area and high electrocatalytic activity and durability for HER applications. What is more, we demonstrate that the loading rate of MoS_2_ nanosheets on graphene has an influence on the HER activity and that the catalytic activity is almost completely unaffected by the various bending states, indicating promising prospects for flexible HER devices.

## Experimental

### Materials synthesis

#### Synthesis of graphene oxide (GO)

GO was made using a modified Hummers’ method. Briefly, 1 g of graphite powder was put into a 250 mL round-bottom flask. A total of 25 mL of concentrated H_2_SO_4_ (98%) was added, and the mixture was stirred for 10 min in an ice water bath. Then, 0.5 g of NaNO_3_ powder was added and stirring continued for 20 min. Next, 3 g of KMnO_4_ was slowly added to the flask, keeping the reaction temperature below 5 °C for 30 min. Then, the solution was heated in an oil bath at 35 °C and allowed to stir for 0.5 h. Afterward, the flask was removed from the oil bath, and 50 mL of water and 5 mL of 30% H_2_O_2_ were added to end the reaction. The obtained dispersion was filtered and washed with 10% HCl aqueous solution followed by ample water to remove metal ions and residual acid, respectively. The resultant GO solution was sonicated for 1 h and centrifuged at 5000 rpm. The concentration of GO was determined by weighing the quantity of GO by vacuum freeze-drying 10 mL of GO solution. The final concentration of GO solution was modulated to 5 mg mL^−1^.

#### Synthesis of MoS_2_/RGO hybrid nanosheets

MoS_2_/GO nanosheets were synthesized through a one-step solvothermal reaction according to Dai’s group’s method.^42^ Briefly, 25 mg of GO was dispersed in 40 mL of dimethylformamide (DMF) solution and stirred for 10 min. Then, different amounts of (NH_4_)_2_MoS_4_ (1.25, 2.5, 5, 7.5 or 10 mg) were added to the mixture, followed by sonicating for 10 min to obtain a well dispersed solution. After that, the solution was transferred into a 50 mL Teflon-lined stainless steel autoclave and sealed for reaction at 210 °C for 12 h. After cooling down, the black products were collected by centrifugation at 8000 rpm for 3 min and washed repeatedly with DI water and ethanol 3 times. Finally, the hybrid suspension was vacuum filtered using polyvinylidene difluoride (PVDF) membranes (0.45 μm in pore size, 2 inches in diameter). The wet MoS_2_/GO composite paper along with the PVDF membrane was fabricated, then washed with ethanol to remove the remaining solvent. After vacuum freeze-drying, the composite paper could be easily peeled of as a freestanding MoS_2_/GO paper. The hybrid papers with various loading amounts of MoS_2_ were denoted by weight percentage content as: MoS_2_/GO-5%, MoS_2_/GO-10%, MoS_2_/GO-20%, MoS_2_/GO-30% and MoS_2_/GO-40%. To obtain highly conductive graphene sheets and highly crystalline MoS_2_, the MoS_2_/GO composite papers were cut into 2 cm × 1 cm pieces, then heat-treated at 700 °C for 2 hours in Ar flow with a heating rate of 6 °C min^−1^. For comparison, pure RGO paper was also prepared *via* the above method without the addition of the (NH_4_)_2_MoS_4_ precursor.

### Characterization

The morphology of the as-obtained products was observed using field emission scanning microscopy (FESEM, JSM-7100F, JEOL). X-ray diffraction (XRD) was carried out from 2*θ* = 10° to 80° using a Bruker Advanced D8 diffractometer under Cu Kα radiation (*λ* = 0.1542 nm), with a voltage of 40 kV and a current of 40 mA. The phase structures were characterized using Raman spectroscopy (Jobin-Yvon HR800), with the excitation source an argon-ion laser (*λ* = 515 nm). Transmission electron microscopy (TEM) and high resolution transmission electron microscopy (HRTEM) images were obtained under an acceleration voltage of 200 kV with a FEI Tecnai G20 TEM instrument. The high resolution energy dispersive X-ray spectroscopy (EDS) mapping measurements were carried out with a probe-Cs-corrected HRTEM instrument (FEI Titan G2 60-300) in scanning TEM mode (STEM). The chemical composition and bonding states were investigated by X-ray photoelectron spectroscopy (XPS, Escalab 250Xi) using an Al Kα source 1486.6 eV anode. All XPS spectra were corrected using the C 1s line at 284.6 eV.

### Electrochemical measurements

All electrochemical experiments were performed in a standard three electrode setup in 0.5 M H_2_SO_4_ at room temperature using an electrochemical workstation (ZENNIUM, ZAHENR). The prepared MoS_2_/graphene freestanding paper was measured as a working electrode. A commercial 20% Pt–C catalyst was used as a reference sample. An Ag/AgCl electrode with saturated KCl solution and a Pt wire were used as the reference electrode and counter electrode, respectively. The potential values reported in this study are *versus* the reversible hydrogen electrode (RHE). The HER activities of the samples were evaluated using linear sweep voltammetry at 10 mV s^−1^. Continuous cyclic voltammetry (CV) was conducted at 50 mV s^−1^ between 0.1 and −0.5 V to investigate the cycling stability. EIS was carried out from 10^5^ to 0.1 Hz with an amplitude of 10 mV at the open-circuit voltage.

## Results and discussion

**Fig. 1:**
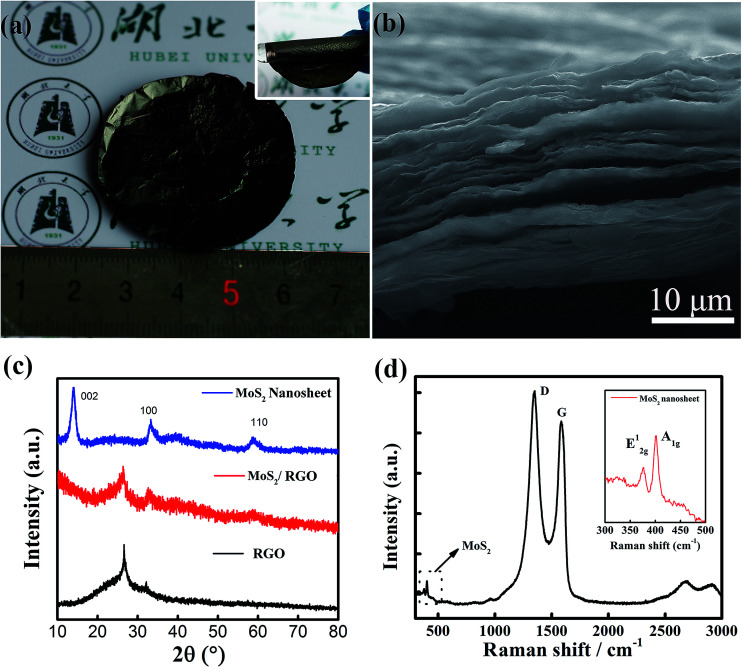
Morphology and phase identification of the MoS_2_/graphene paper. (a) Photograph of a 2-inch free-standing flexible MoS_2_/RGO thin film. The inset shows the flexibility of the hybrid film. (b) SEM image of a cross-section of MoS_2_/RGO film made by fracturing a sample. (c) XRD patterns and (d) Raman spectra of the MoS_2_/RGO hybrid thin films.

We employed a one step solvothermal reaction method to synthesize the MoS_2_/graphene nanocomposites and prepare the free-standing MoS_2_/graphene electrode paper using a vacuum filtration method, which was a facile and time-saving method. Fig. 1a is a typical photograph of the prepared MoS_2_/graphene freestanding paper after peeling off the membrane, with a size of 2 inches. The hybrid film appeared uniform and was a dark gray color. These freestanding papers can be randomly wrapped around a glass rod with no noticeable damage, indicating their remarkably flexible and durable properties (inset in Fig. 1a). Fig. 1b presents the cross-sectional SEM image. The MoS_2_/graphene layers stack together to form a compact layered structure with a thickness of about 20 μm due to van der Waals forces. The MoS_2_/graphene hybrid was characterized by XRD as shown in Fig. 1c. For comparison, pure RGO films and MoS_2_ nanosheet samples were evaluated too. The RGO films show a broad peak at 26.1°, which indicated the stacking structure of the RGO sheet. The pure MoS_2_ nanosheet as prepared by the same method shows three broad diffraction peaks indicating nanosize MoS_2_ crystal domains with a hexagonal structure (JCPDS: 65-0160). The hybrid MoS_2_/RGO films show two diffraction peaks relating to RGO and MoS_2_ (100), respectively. The absence of a (002) low-angle diffraction peak indicates that the atomic arrangement of MoS_2_ in the hybrid with graphene sheets was different from that in the powder form. Raman spectroscopy, as shown in Fig. 1d, revealed the characteristic D and G bands of graphene, and the peaks of MoS_2_ at 378 and 402 cm^−1^, corresponding to the E_2g_^1^ and A_1g_ modes as shown in the inset, respectively. The lower intensity of E_2g_^1^ (in-plane Mo–S phonon mode) compared to that of A_1g_ (out-of plane Mo–S phonon mode) indicates that the MoS_2_ crystals tended to show the basal-edge-rich feature of the ultrathin plate growth, in good agreement with the absence of a (002) peak in the XRD measurements.

**Fig. 2:**
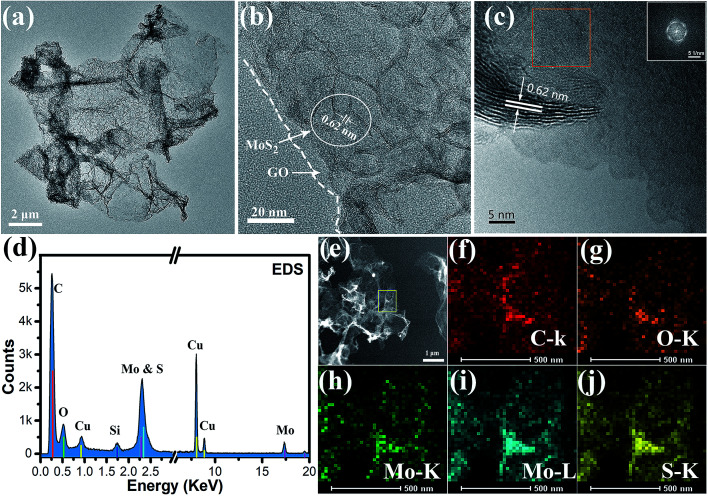
TEM characterization of MoS_2_ nanosheets on graphene oxide film. (a and b) Low resolution TEM images of the MoS_2_/GO hybrid and TEM image showing the folded edges of the MoS_2_ nanosheet. (c) High resolution image of horizontally aligned (basal plane exposed) and vertically aligned (edge exposed) MoS_2_ nanosheets. The inset shows the FFT image of the red square area. The vertically aligned layer-to-layer spacing is 0.62 nm. (d) TEM EDX spectrum of the MoS_2_/GO hybrid. (e) STEM and (f–j) STEM-EDS mapping images of C, O, Mo and S elements in the MoS_2_/GO nanosheets.

The morphology, microstructure and chemical element information for the ultra thin plate MoS_2_/graphene nanosheets were further obtained by TEM. As shown in Fig. 2a, the low resolution TEM image verifies that the ultrathin MoS_2_ nanosheets are homogeneously dispersed on the graphene sheets, indicating that the graphene sheets could be used as an efficient substrate for the nucleation and growth of MoS_2_ nanosheets. On the other hand, the graphene sheets show good ability to prevent the aggregation of MoS_2_ nanosheets, serving to enlarge the active area of HER. Furthermore, plate-like ultrathin MoS_2_ can be further verified by HRTEM, where each sheet contains several S–Mo–S layers with an interlayer spacing of 0.62 nm, as shown in Fig. 2b. Dense interconnected ripples and corrugations can also be observed, suggesting the basal-edge-rich feature of the ultrathin MoS_2_ nanosheet, in agreement with the results of XRD and Raman characterization. Furthermore, two different aligned (horizontally aligned and vertically aligned) MoS_2_ nanosheets were found on the GO sheet under higher resolution TEM investigations, as shown in Fig. 2c. The HRTEM image and a selected area (red square) electron diffraction pattern (inset) of horizontally aligned (basal plane exposed) MoS_2_ show that a few layers of MoS_2_ were stacked with hexagonal lattice symmetry. Part of the MoS_2_ layer is fully vertically aligned, resulting in dominantly exposed molybdenum or sulfur atoms, which is beneficial for higher chemical catalytic activity for the MoS_2_ sheets. The HRTEM image indicates the vertically aligned MoS_2_ composed of a several-layer structure and a lattice fringe spacing of 0.62 nm. The chemical composition of the hybrid nanosheet was further determined by an energy dispersive X-ray spectroscopy (EDS) curve and mapping using a probe-Cs-corrected TEM instrument. Fig. 2d shows the EDX spectrum of the MoS_2_/GO hybrid films. The results show that there were six kinds of element (Cu, Si, C, O, Mo and S) detected, of which C and O peaks come from GO, Mo and S come from MoS_2_, and Cu and Si come from the TEM grid and silicon escape peak. Fig. 2e–j show the STEM and STEM-EDS mapping images of C, O, Mo and S elements. It is obvious that all of the Mo, S and C elements uniformly distribute on the whole nanosheet surface. This further verified the formation of the as-predicted MoS_2_/graphene nanosheet hybrid structure. The signal for O comes from the incomplete reduction of graphene oxide after the solvothermal reaction. To obtain highly conductive graphene sheets, the freestanding MoS_2_/graphene paper was further treated by annealing at 700 °C for 2 h in Ar flow.

**Fig. 3:**
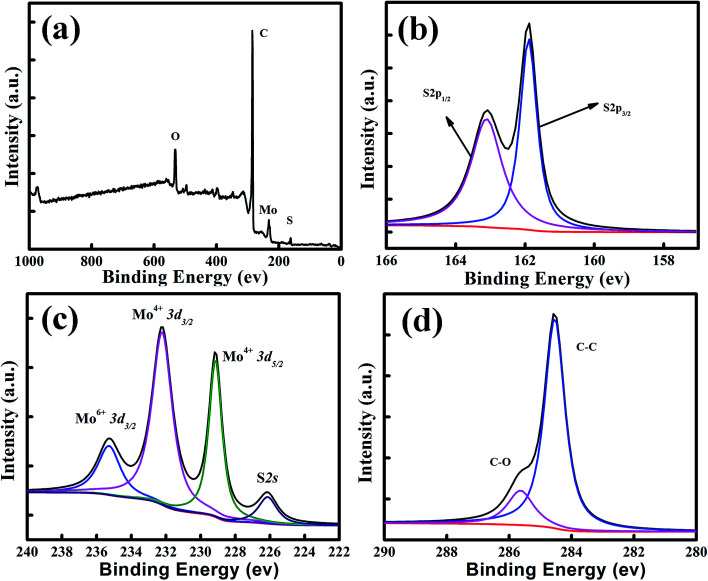
Composition characterization of the MoS_2_/graphene films. (a) XPS survey scan, and high-resolution scans of (b) S 2p, (c) Mo 3d and (d) C 1s electrons of the MoS_2_/graphene hybrid.

X-ray photoelectron spectroscopy (XPS) analyses of the annealed MoS_2_/graphene catalyst films were carried out to elucidate the chemical bonding structures. A typical XPS survey scanning in the binding energy range from 0 to 1200 eV is shown in Fig. 3a. For the MoS_2_/graphene hybrid film, Mo, S, O and C elements were detected. Meanwhile, the S 2p region (Fig. 3b) exhibits primarily a single doublet with the S 2p_3/2_ peak at 161.7 eV and the S 2p_1/2_ peak at 163.1 eV, which is consistent with the −2 oxidation state of sulfur. As shown in Fig. 3c, two characteristic peaks arising from Mo 3d_5/2_ and Mo 3d_3/2_ orbitals are located at 229 and 231.1 eV, suggesting the dominance of Mo^4+^ in the nanosheet. In addition, a lower peak located at 236 eV was also detected, which was attributed to Mo–O bonding at the surface of the RGO nanosheet. The oxidation degrees of the RGO sheets could be evaluated using the area ratios of the C 1s XPS peaks of oxidized (*A*_CO_) and intact (*A*_CC_) carbon atoms, as shown in Fig. 3d. The C 1s peaks were deconvoluted into two peaks that correspond to the sp^2^ carbon (C=C, 284.3 eV) and epoxy/hydroxyls (C–O, 285.4 eV), respectively. The ratio *A*_CO_/*A*_CC_ of annealed MoS_2_/graphene is about 0.25, which is much lower than that of the GO nanosheet published elsewhere,^43^ indicating that the GO sheet has been reduced to RGO efficiently by annealing.

**Fig. 4:**
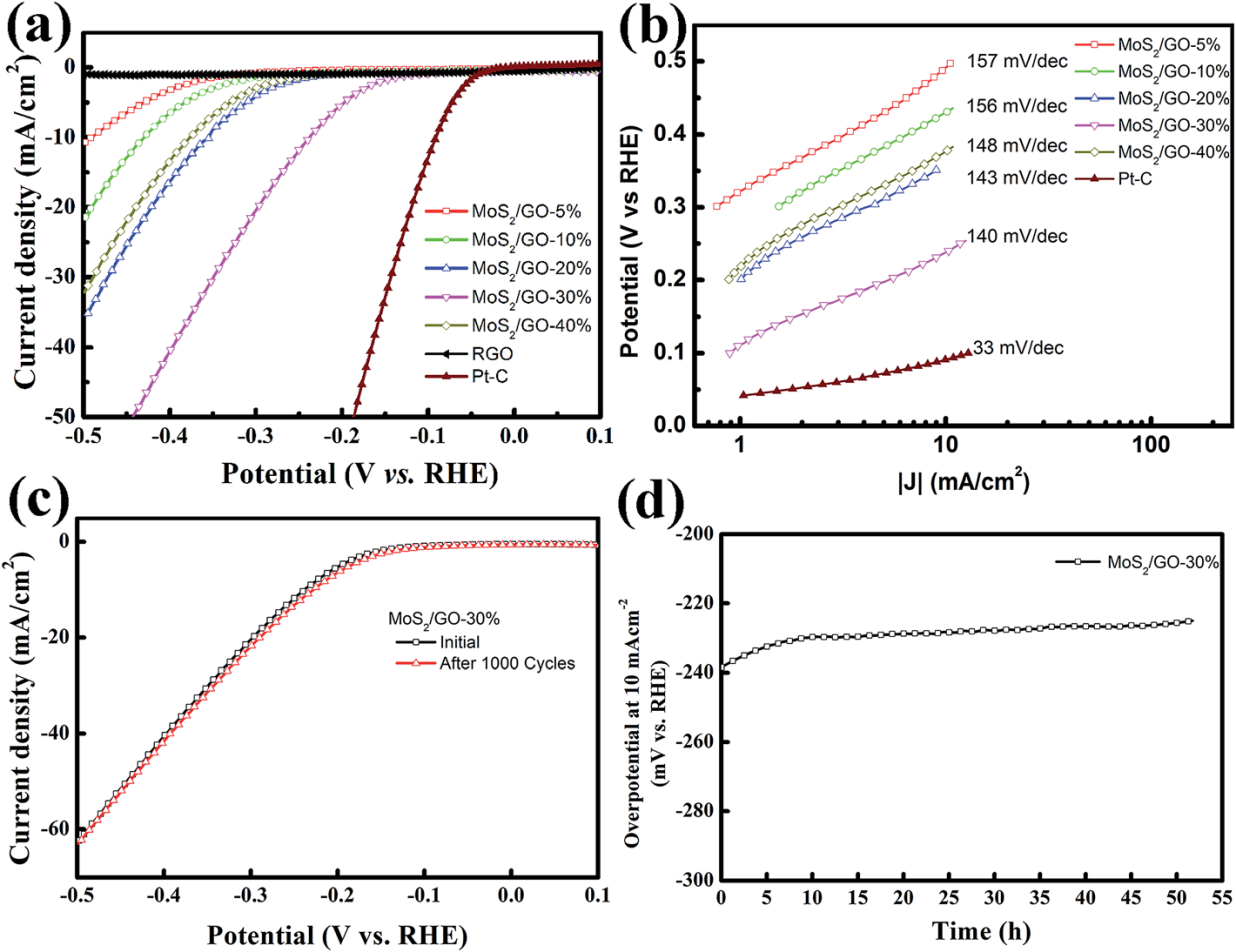
HER activity characterization. (a and b) Polarization curves and Tafel plots of MoS_2_/graphene films with various loading rates from 5% to 40%. Pure RGO film and a commercial Pt–C electrode were measured as references. (c and d) Durability test for the MoS_2_/GO-30% catalyst with 1000 cycles and a fixed static polarization current of 10 mA cm^−2^ for over 50 hours.

As proven by computational and experimental studies, the chemically active undercoordinated atoms at the edge sites of MoS_2_ play a key role in improving the HER activity.^29,44^ Therefore, efficient HER activity can be expected for the higher loading rate of MoS_2_ in the hybrid films, which introduced more edge sites. We investigated the HER activities of our MoS_2_/graphene hybrid thin films with different loading rates. The electrocatalytic HER performance of freestanding MoS_2_/graphene paper was measured in 0.5 M H_2_SO_4_ solution using linear sweep voltammetry (LSV) in a three-electrode system. The polarization curve was recorded within a cathodic potential window in the range 0.1 to −0.5 V at a 10 mV s^−1^ scan rate. For comparison, pure RGO film and commercial Pt–C were also assessed. Fig. 4a shows the corresponding polarization curves for the MoS_2_/graphene hybrid films loaded with various MoS_2_ nanosheet percentages from 5 to 40%. All the polarization curves have no IR compensation. As expected, the commercial Pt–C catalyst displayed the highest electrocatalytic activity, with an onset overpotential (defined here as the overpotential at which the HER current density is 1.0 mA cm^−2^) of 23.9 mV. In sharp contrast, the pure RGO film without MoS_2_ nanosheets exhibited the lowest electrocatalytic activity. The onset overpotential gradually decreased from 263.7 mV to 94.2 mV when the MoS_2_ nanosheet content percentage increased from 5% to 30%. However, in contrast the overpotential increased to 221.6 mV when the MoS_2_ nanosheet percentage was 40%. Generally, the overpotential value for a HER current density of 10 mA cm^−2^ is an important reference because significant hydrogen evolution can be observed and the solar-light-coupled HER apparatuses usually operate at 10–20 mA cm^−2^ under standard conditions (1 sun, AM 1.5).^45^ According to a previous report, monolayer MoS_2_ loaded on a 3D nanoporous gold substrate shows a low operating voltage of 226 mV as a typical overpotential for a few-layer MoS_2_ nanosheet.^29^ To achieve this current density in this work, the 5 wt%-MoS_2_/GO catalyst requires an overpotential of 490.2 mV. Moreover, the overpotential shows an obvious decrease from 430.3 mV to 238.5 mV when the weight percentage content of MoS_2_ nanosheets increases from 10% to 30%, which indicates that the MoS_2_ nanosheets have significantly accelerated the HER process. However, the overpotential was remarkably increased from the lowest value 238.5 mV to 377.2 mV, as the MoS_2_ loading rate increases from 30% to 40% in the MoS_2_/GO hybrid thin films, which indicated that the HER activity was suppressed in the catalyst in spite of the higher loading rate bringing more active sites. We speculate that the surface loading rate of the RGO film was the major reason for the HER activity performance degradation. Part of the MoS_2_ nanosheets were interstitial in the RGO hybrid thin films when the concentration of the MoS_2_ precursor was higher than the loading capacity of the GO sheets. This may lead to inefficient electron transfer because of the semiconducting properties of the S–Mo–S layers. For comparison, *I*–*V* curve measurements of the thin hybrid film with various loading rates were carried out to elucidate their electrical resistivity (ESI Fig. S2†). These data verified that the excellent catalytic activity of the thin MoS_2_/GO hybrid films may arise from the synergetic effect of the abundant active edge sites and efficient electron transfer. To obtain further insight into the HER properties of the various catalysts, the Tafel slopes were investigated. The linear portions of the Tafel plots were fit to the Tafel equation, yielding Tafel slopes of 156.7, 156.1, 142.6, 140.1 and 147.8 mV dec^−1^ for 5%, 10%, 20%, 30% and 40% MoS_2_ loading rate on RGO thin films, as shown in Fig. 4b, respectively. These results are in agreement with overpotential investigations (ESI Table 1†). Electrochemical impedance spectroscopy was performed under HER conditions. The Nyquist plots are shown in ESI Fig. S3† and reveal the charge transfer resistance for MoS_2_ and RGO hybrid nanosheets.

The stability of the MoS_2_/graphene nanosheets catalyst was evaluated by cycling the electrode at 50 mV s^−1^ between 0.1 and −0.5 V in 0.5 M H_2_SO_4_ solution at room temperature. The performance of the MoS_2_/GO-30% catalyst after 1000 cycles is shown in Fig. 4c. As observed, the polarization curve for the MoS_2_/GO hybrid nanosheet catalyst remained almost the same after 1000 cycles. In addition, the durability of the MoS_2_/GO-30% catalyst was also examined by electrolysis at a static polarization current of 10 mA cm^−2^ for over 50 hours, as shown in Fig. 4d. The overpotential at a current density of 10 mA cm^−2^ decreased by a relatively small value (from 238 to 225 mV) after 50 hours of durability determination, indicating that MoS_2_/graphene nanosheet catalysts are stable electrocatalysts in acidic solution.

**Fig. 5:**
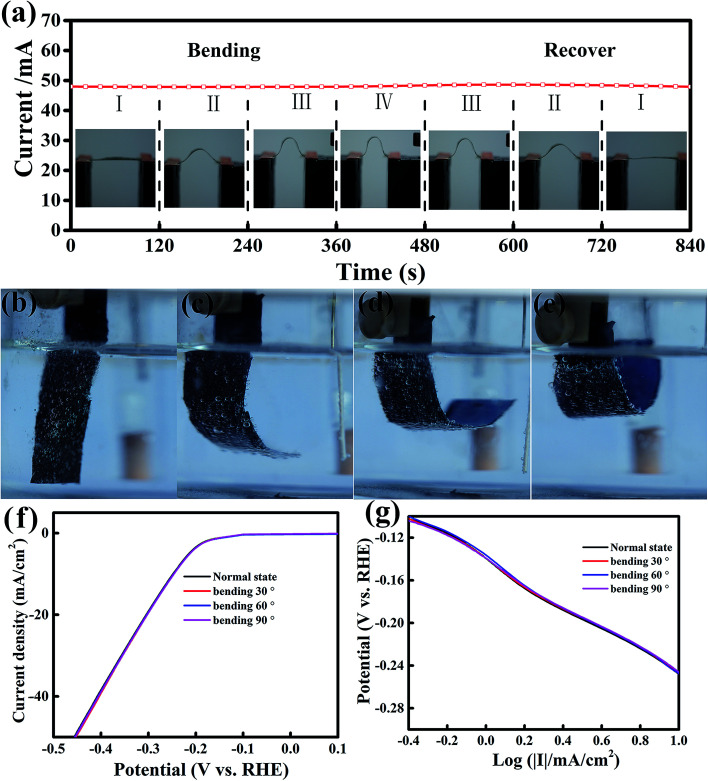
The mechanical flexibility of MoS_2_/graphene films. (a) *I*–*t* curve of the MoS_2_/graphene film bent with various curvatures under a constant voltage of 1 V. (b–e) Optical photographs of the MoS_2_/graphene electrode operated under four various bending states. (f and g) Polarization curves and fitted derivative polarization curves in the linear region of the MoS_2_/graphene film with various bending states.

Recently, flexible and wearable electronic devices and energy storage and conversion systems, such as flexible displays, supercapacitors and solar cells, have attracted great attention.^46^ Therefore, it is highly interesting and urgent to investigate flexible electrocatalysts to develop a flexible power source with remarkable electrochemical performance for integration into flexible electronics.^47^ To develop flexible electrocatalysts, the catalytic performance should remain unchanged under various bending states. The current stability of MoS_2_/RGO hybrid films under four various bending curvatures was monitored at a fixed voltage of 1 V (Fig. 5a), and each bending curvature was maintained for 120 s. We found that the current was very stable and had no apparent change under various bending and recovering states, indicating that the conductance of the MoS_2_/RGO catalyst was hardly affected by bending stress. Furthermore, the effect of the flexibility of MoS_2_/RGO on catalytic activity was investigated at four various bending states, as shown in Fig. 5b–e, which were measured in 0.5 M H_2_SO_4_ acidic solution at 10 mV s^−1^. It is clear to see from Fig. 5f that the polarization curve almost stays the same under the various bending states. In addition, the derivative polarization curves under four various bending states are shown in Fig. 5g, which also shows that the derivative polarization curve fits a linear regression under various bending states. Therefore, the above results indicate that MoS_2_/RGO is an excellent flexible electrocatalyst and its electrocatalytic activity is almost unaffected by the various bending states.

## Conclusion

In conclusion, we have demonstrated a one-step *in situ* solvothermal process for the fabrication of improved flexible hybrid MoS_2_/graphene free-standing electrocatalyst paper for HER applications. The MoS_2_/graphene catalysts exhibit significantly enhanced electrocatalytic activity and cycling stability towards the splitting of water in acidic solution, indicating the promising potential of solar energy. Furthermore, a strategic balance of abundant active edge sites with efficient electron transfer in the MoS_2_/graphene hybrid catalyst plays a key role in controlling the electrochemical performance of the MoS_2_ nanosheets. Most importantly, because of invariant performance under various bending states, the MoS_2_/graphene hybrid films provide a fundamental opportunity for the development of flexible electrocatalysts, which have potential technological applications in renewable energy conversion and energy storage systems.

## Conflicts of interest

There are no conflicts to declare.

## Supplementary Material

RA-008-C8RA01226A-s001
