# Supplementary material for: In situ synthesis of MoS2/graphene nanosheets as free-standing and flexible electrode paper for high-efficiency hydrogen evolution reaction
Source: RSC Adv. 2018 Mar 16;8(19):10698–705. doi: 10.1039/c8ra01226a (PMC9078904; doi:10.1039/c8ra01226a)
Supplement: RA-008-C8RA01226A-s001 [file RA-008-C8RA01226A-s001.pdf]

Electronic Supplementary Material (ESI) for RSC Advances.
This journal is © The Royal Society of Chemistry 2018

Supporting information:

In-situ Synthesis of MoS_2/graphene Nanosheets as Free-standing and Flexible Electrode Paper for High-Efficiency Hydrogen Evolution Reaction

Xianghui Zhang [1*], Mingguang Zhang [1], Yiqun Tian [1], Jing You [1], Congxing Yang [2], Jun Su [2], Yuebin Li [1], Yihua Gao[2], and Haoshuang Gu [1*]

1. Hubei Collaborative Innovation Center for Advanced Organic Chemical Materials, Hubei Key Laboratory of Ferro & Piezoelectric Materials and Devices, Faculty of Physics & Electronic Sciences, Hubei University, Wuhan, 430062, P.R. China.
2. Center for Nanoscale Characterization and Devices, School of Physics and Wuhan National Laboratory for Optoelectronics, Huazhong University of Science and Technology , Luoyu Road 1037, Wuhan 430074, P.R. China.

Email： xhzhang@hubu.edu.cn, guhsh@hubu.edu.cn

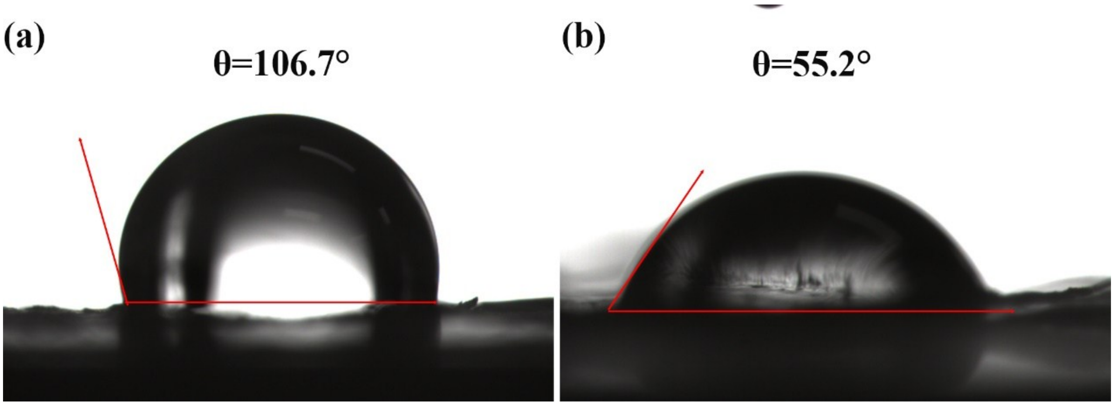

Figure S1 Contact angle measurement of MoS_2/graphene hybrid film (a) before and (b) after heat-treated at 700 ^oC for 2 hours in Ar flow.

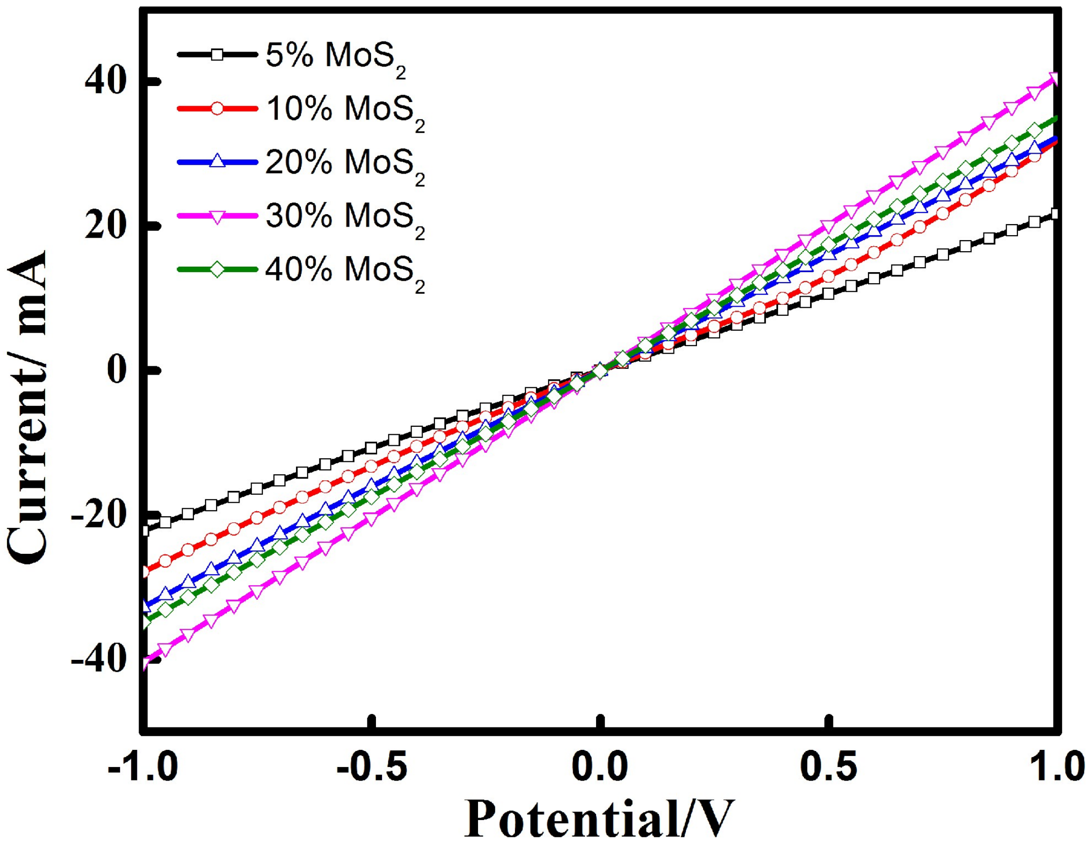

Figure S2. The I-V curve measurement of the MoS_2/graphene hybrid thin films with various loading rate from 5% to 40%.

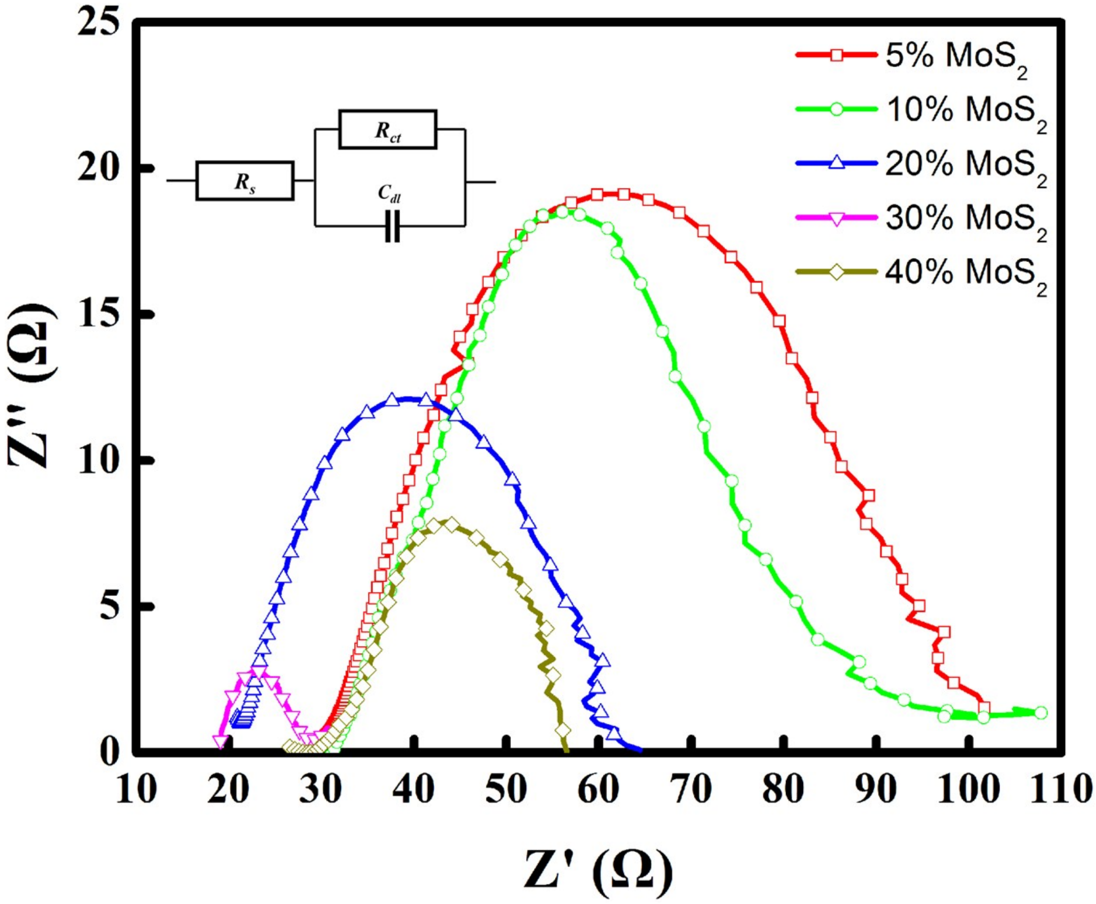

Figure S3. The Nyquist plots of MoS_2/graphene hybrid thin films with various loading rate collected from 10^5 to 0.1 Hz with an amplitude of 10 mV at the open-circuit voltage.

Table 1. Comparison of catalytic parameters of various loading rate MoS_2 catalysts and commercial Pt-C catalyst.

Catalyst	Onset overpotentials (mV vs RHE)	Tafel slopes (mV/dec)	Exchange current density ($\mu A/cm^2$)	η(mV) @j=10 mA/cm^2
5% MoS_2	263.7	156.7	10.3	490.2
10% MoS_2	261.3	156.1	18.6	430.3
20% MoS_2	212.1	142.6	33.7	355.5
30% MoS_2	94.2	140.1	168.4	238.5
40% MoS_2	221.6	147.8	27.5	377.2
Pt-C	23.9	33.9	51.6	91.2

Table 2. Electrochemical Impedance Spectroscopy (EIS) parameters of different loading rate MoS_2 catalysts

Catalyst	Double-layer capacitance (μF)	Charge-transfer Resistance (R_{ct}/Ω)	Series Resistance (R_s/Ω)
5% MoS_2	379.8	83.5	6.2
10% MoS_2	216.7	36.6	31.6
20% MoS_2	108.5	35.5	26.5
30% MoS_2	92.2	3.8	36.6
40% MoS_2	85.6	14.5	30.6
